# Functional MRI in Peripheral Arterial Disease: Arterial Peak Flow versus Ankle-Brachial Index

**DOI:** 10.1371/journal.pone.0088471

**Published:** 2014-02-05

**Authors:** Bas Versluis, Patty J. Nelemans, Rutger Brans, Joachim E. Wildberger, Geert-Willem Schurink, Tim Leiner, Walter H. Backes

**Affiliations:** 1 Maastricht University Medical Center, Departments of Radiology, Maastricht, The Netherlands; 2 Maastricht University Medical Center, Department of Surgery, Maastricht, The Netherlands; 3 Maastricht University Medical Center, Department of Epidemiology, Maastricht, The Netherlands; 4 Cardiovascular Research Institute Maastricht (CARIM), Maastricht, Netherlands; Van Andel Research Institute, United States of America

## Abstract

**Objectives:**

The purpose of this study was to compare the success rate of successful arterial peak flow (APF) and ankle-brachial index (ABI) measurements in patients with suspected or known peripheral arterial disease (PAD).

**Materials and Methods:**

183 patients with varying degrees of PAD were included. All subjects underwent ABI measurements and MR imaging of the popliteal artery to determine APF. Proportions of patients with successful APF and ABI measurements were compared and the discriminative capability was evaluated.

**Results:**

APF was successfully measured in 91% of the patients, whereas the ABI could be determined in 71% of the patients (p<0.01). Success rates of APF and ABI were significantly higher in patients with intermittent claudication (95% and 80%, respectively) than in patients with critical ischemia (87% and 62%, respectively).

**Conclusions:**

Compared to the assessment of PAD severity with ABI, the success rate of MRI-based APF measurements in patients with a clinical indication for MRA is 20% higher, with similar discriminatory capacity for disease severity. Therefore, APF is an especially convenient and valuable measure to assess severity in PAD patients scheduled to undergo MR angiography to obtain additional functional information concerning the vascular status.

## Introduction

Peripheral arterial disease (PAD) is a highly prevalent condition in industrialized societies [1], [2], affecting up to 7% of the general population over the age of 70 years [2]. The diagnosis of PAD is based on the typical clinical history, physical examination with palpation of pedal pulse and by measuring the ankle-brachial index (ABI) [3], [4], [5], [6]. The ABI is a fast, widely available and cost-effective test for this purpose [7], [8], [9], [10], [11], [12]. Sensitivity of ABI measurements for diagnosing PAD is high and the ABI can be used to determine the severity and progression of PAD over time [11], [13], [14], [15]. Unfortunately, ABI measurements also suffer from certain limitations such as inability to reliably acquire the ABI in many patients with stiff and uncompressible ankle arteries due to severe arterial wall calcifications and poor interobserver variability [16], [17], [18]. For example, studies have shown that the ABI cannot be measured in at least 5 – 10% of the diabetic patients [12], [15], [18], [19], [20], [21], [22], [23].

In addition to ABI measurements, diagnostic (imaging) tools such as duplex ultrasonography (DUS), magnetic resonance angiography (MRA), computed tomography angiography (CTA) or invasive angiography are available for diagnosis, treatment planning and longitudinal follow-up of patients with PAD [2]. Except DUS, all of these imaging modalities are accurate for visualizing the vascular morphology [24], [25], [26], but generally do not provide functional information, which makes it difficult to quantify and evaluate hemodynamic consequences over time, as is easily done by ABI measurements. MRI, however, can also be used to evaluate vascular function, for example by measuring arterial peak flow (APF) with quantitative MR phase-contrast imaging [27]. The APF is a highly reproducible [27], [28], fast and simple measurement, which is not hampered by uncompressible arteries like ABI measurements. For those PAD patients scheduled to undergo a MRA examination and in whom the ABI cannot be determined, the combination of morphologic MRA and functional vascular MRI might help in the objective assessment of vascular status and be helpful in the follow-up of PAD patients, especially as due to the progressive character of PAD many patients will repeatedly undergo MRA examinations during lifetime. APF measurements in that respect are most promising if the proportion of successful APF measurements is markedly higher as compared to ABI measurements in this group of patients with PAD.

Therefore, the purpose of this study was to compare the proportions of a large group of patients scheduled for MRA with successful APF and ABI measurements. In addition, we determined the influence of disease severity (intermittent claudication versus critical ischemia), presence of diabetes mellitus and the location of arterial lesions on the success rates of APF and ABI measurements. Also the discriminative capability for disease severity of the APF and ABI measurements was determined.

## Materials and Methods

### Study population

This study was approved by the institutional Medical EThics Committee Academic Hospital Maastricht/University Maastricht (METC azM/UM). Considering the retrospective nature of the study the ethics committee waived the need for informed consent. The APF is routinely measured at our institution in every patient scheduled for contrast-enhanced MR angiography (CE-MRA) of the peripheral arteries. Therefore, 210 consecutive patients undergoing CE-MRA of the peripheral arteries at our institution as part of a clinical routine examination between January and June 2010 were considered suitable for analysis in this study. In 27 patients CE-MRA was performed to rule out deep venous thrombosis (n = 16), or as part of the pre-operative visualization of the vasculature in patients scheduled for reconstructive surgery, with the lower leg serving as donor site (n = 11). After exclusion of these 27 patients, who did not undergo ABI measurements, 183 patients were further analyzed in this study. Patient characteristics are presented in table 1. Distinction between intermittent claudication and critical ischemia was based on clinical symptoms as recorded by the referring vascular surgeon and retrieved from patient records [7].

**Table 1:** Patient characteristics.

	Intermittent claudication	Critical ischemia	All patients
**Patients**	94	89	183
**Males (%)/Females (%)**	58 (62%)/36 (38%)	45 (51%)/44 (49%)	103 (56%)/80 (44%)
**Age (mean ± SD years)**	64±12	69±11	67±12
**Diabetics (%)**	42 (48%)^a^	38 (48%)^b^	80 (48%)
**Symptomatic leg:**			
**Left**	34	31	65
**Right**	33	44	77
**Both**	27	14	41
**Total**	121	103	224
			
**APF (mL/s)**	5.8±3.1	3.9±2.8^a^	4.9±3.1
**ABI**	74±22	52±24^a^	65±25

APF, arterial peak flow; ABI, ankle-brachial index.

a Diabetic status was confirmed in 87 out of 94 patients.

b Diabetic status was confirmed in 79 out of 89 patients.

### MRI protocol

**Figure 1:**
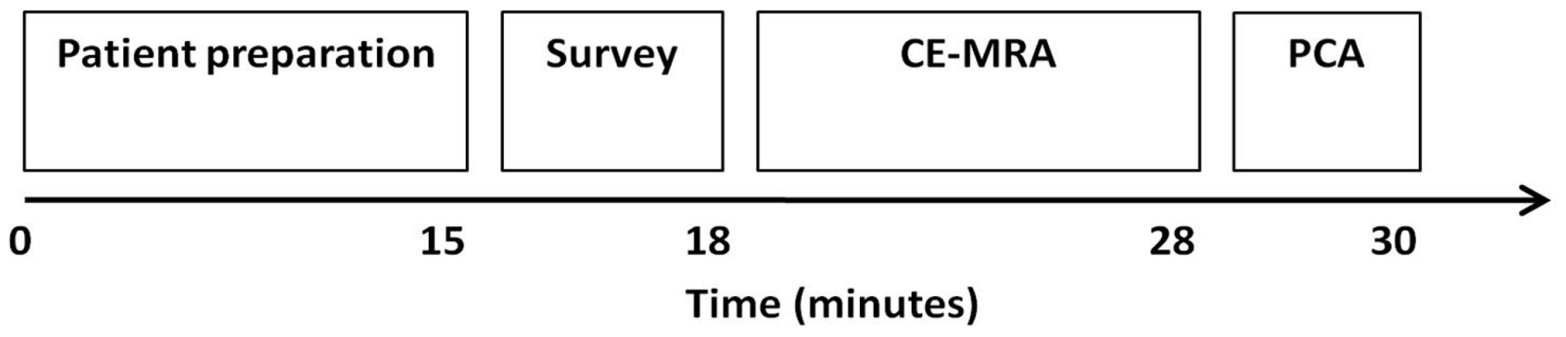
Overview of imaging protocol and the approximate duration of each acquired sequence. Contrast-enhanced MR angiography (CE-MRA) was combined with phase-contrast angiography (PCA) flow MRI. Total MRI examination time, including patient positioning, table movements and MRI scans, was approximately 30 minutes.

All patients underwent quantitative cine PCA flow measurement of the popliteal artery (PA) to obtain flow wave forms, as required to determine the APF [29]. PCA was preceded by a three-station CE-MRA protocol of the peripheral arteries. A schematic overview of the scan protocol is given in figure 1. All examinations were performed on a 1.5-T MRI system (Intera, Philips Medical Systems, Best, The Netherlands). Patients were imaged in supine position and had been in this position for approximately 30 minutes before the flow measurement was started. During this time the CE-MRA was acquired, using a fixed dose of 10 mL gadofosveset trisodium (Ablavar®, Lantheus Medical Imaging, Billerica, MA) as contrast agent.

*MR angiography.* A three-station 3D gradient-echo (fast field echo) MRA sequence was performed as previously described [25], [30]. Acquisition parameters were as follows: TR 4.8 ms, TE 1.45 ms, flip angle 40°, FOV 470 mm, matrix 480, and voxel dimensions (reconstructed) 0.92×0.92×1.20 mm. Prior to contrast agent administration, a non-enhanced ‘mask’ image data set was acquired with exactly the same acquisition parameters as the CE-MRA, enabling background tissue suppression by image subtraction.

*Flow MRI.* For quantitative cine phase-contrast imaging we used a 2D gradient-echo (fast field echo) scan technique with the following acquisition parameters: TR 9.7 ms, TE 5.8 ms, flip angle 30°, FOV 380 mm, matrix 384, and reconstructed voxel dimensions of 0.99×0.99×6.00 mm. Fifteen dynamic phases were acquired to obtain the waveform that spanned the cardiac cycle. The phase encoding velocity was set to 100 cm/s in the craniocaudal direction [27], [29]. Vector cardiography (VCG) triggering was used for retrospective cardiac synchronization. Parallel imaging (sensitivity encoding, SENSE) was applied to reduce scan time (SENSE acceleration factor 2 in the anterior-posterior direction) [31]. At a mean heart rate of 60 beats per minute, nominal acquisition time was 1 minute.

### Angiographic reading

All angiographic datasets were analyzed by one well-trained radiologist with over 5 years of experience in CE-MRA of the peripheral vasculature. For this study, CE-MRA data sets were used to determine whether or not obstructive arterial lesions were present within the peripheral vascular tree of most symptomatic extremity.

### Flow analysis

Modulus and phase images were reconstructed from the cine phase-contrast data. A quantitative flow analysis package included with the software release (QFlow, R11.4.14) of the MRI hardware was used for analysis of the flow waveform.

Using this software, a region of interest (ROI) covering the entire visible cross-section of the artery of interest was accurately drawn manually using a reconstructed modulus image during peak systole and then automatically propagated to the remaining cardiac phases using an active contour algorithm. Although the peak systolic phase is sufficient to calculate the APF, ROI's were propagated to all cardiac phases to obtain flow wave forms, which were visually analyzed to reassure the chosen cardiac phase indeed was at peak systole and to detect possible aliasing effects directly after the acquisition. If detected, the measurement was repeated with sufficient higher phase encoding velocity.

APF was preferred over mean flow for analysis, as peak flow is known to be more reproducible and may exhibit large differences between patients and healthy controls [29]. APF measurements proved highly reproducible before [27], therefore all APF measurements were analyzed by one single blinded, well-experienced radiologist.

### ABI measurements

ABI data were retrieved from patient records. All ABI measurements were performed by well-trained operators at our vascular function laboratory, using an automated Doppler system (Nicolet VasoGuard, VIASYS healthcare, Madison, WI). If the ABI could not be determined due to arterial stiffness or other circumstances, this was mentioned in the report by the operators. The ABI was acquired within 2 months prior to the MRI exam for all patients, and patients did not receive invasive treatment between the two exams.

### Statistical analysis

For statistical analysis only APF and ABI data obtained in the (most) symptomatic leg were used. If both legs were equally symptomatic, the right leg was analyzed. Statistical analysis was performed with commercially available statistical software (SPSS 16.0, SPSS Inc., Chicago, IL). The proportions of patients with successful APF and ABI measurements were compared and tested for statistical significance using McNemar's test for paired proportions. A chi-square test was used to test between group differences in proportions and a *t*-test for independent samples was used to test between-group differences in continuous variables.

The ability of APF and ABI measurements to discriminate between patients with intermittent claudication and critical ischemia was compared using receiver operating characteristic (ROC) curves and the area under the curve (AUC). Differences in AUCs were tested for statistical significance using the method described by Hanley et al, which accounts for the fact that AUCs were derived from the same sample of patients [32]. In all analysis, p-values <0.05 were considered to indicate statistical significance.

## Results

### APF and ABI

**Figure 2:**
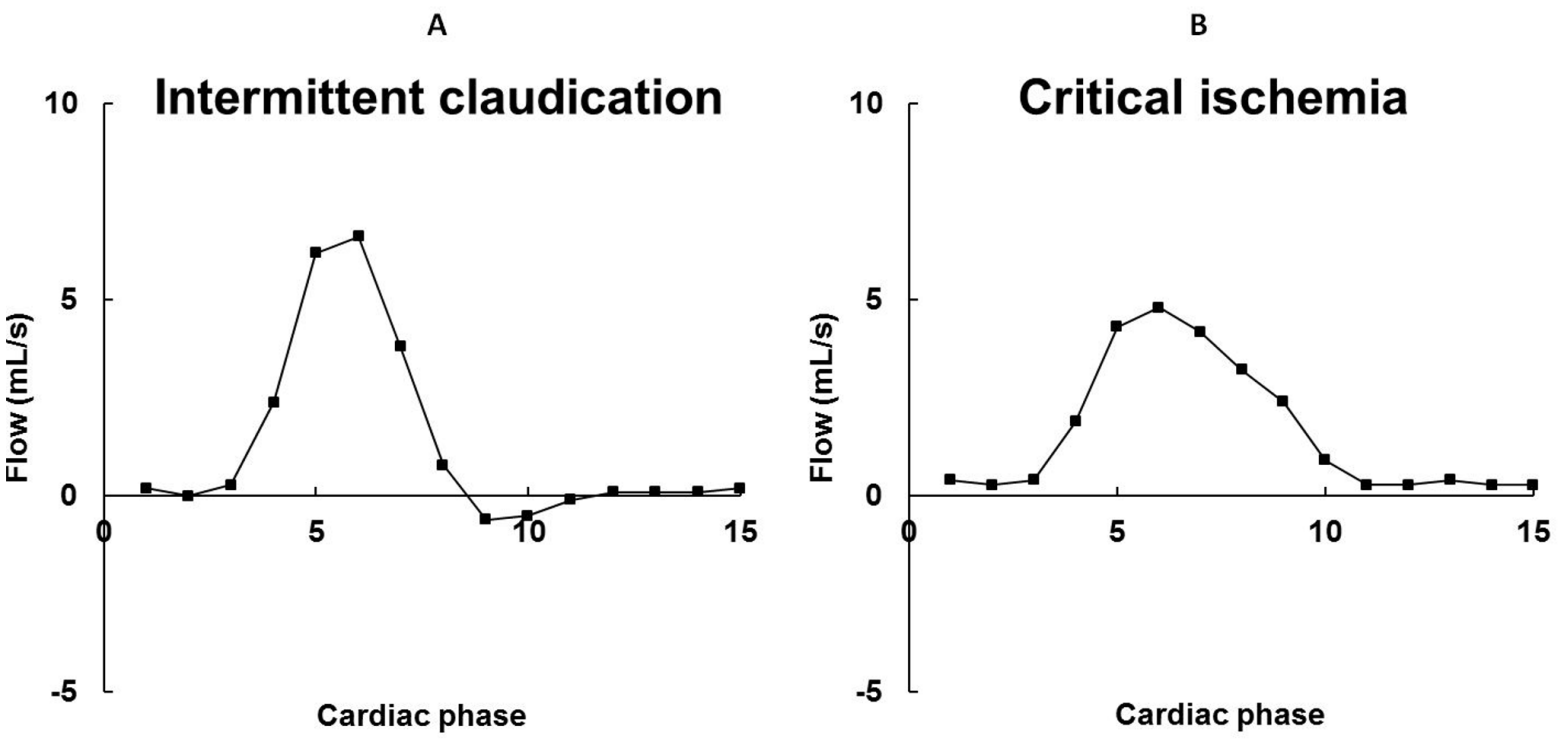
Flow waveforms in patients with intermittent claudication (A) and critical ischemia (B). Note the bi-phasic flow waveform and a higher peak flow value in intermittent claudication compared to the mono-phasic flow waveform with a lower peak flow value in critical ischemia.

The APF measured in the (most) symptomatic leg of 183 patients was 4.9±3.1 mL/s (mean ± SD) and mean ABI was 0.65±0.25 (table 1). Typical flow waveforms of patients with intermittent claudication and critical ischemia are presented in figure 2. Both the APF and ABI were significantly lower in patients with critical ischemia compared to patients with intermittent claudication (p<0.01). In patients with obstructive arterial lesions below the level of the APF measurement (n = 103), APF was significantly lower (3.9±2.7 mL/s) compared to patients without arterial lesions of the lower leg (5.2±3.3 mL/s; p<0.01). Comparable differences were found for the ABI measurements (0.56±0.23 and 0.68±0.25, respectively; p<0.01).

### Success rates of APF and ABI measurements

Success rates of APF and ABI measurements are presented in tables 2 and 3. Success rates of APF measurements were significantly higher compared to ABI measurements (91% versus 71%, p<0.01). APF and ABI measurements were successful with both methods in 121 (66%) patients, whereas in 96% of the patients either APF or ABI measurements were successful. In 45 (25%) patients only the APF could be determined and in 9 (5%) patients only the ABI could be determined.

**Table 2:** Success rates of APF and ABI measurements.

	Successful APF	Successful ABI	p-value
**All patients**	91% (166/183)	71% (130/183)	<0.01
			
**Disease severity:**			
**IC**	95% (89/94)	80% (75/94)	<0.01
**CI**	87% (77/89)	62% (55/89)	<0.01
			
**Diabetes** a			
**Yes**	95% (76/80)	69% (55/80)	<0.01
**No**	90% (77/86)	71% (61/86)	<0.01
			
**Obstruction below PA** b			
**Yes**	88% (91/103)	68% (70/103)	<0.01
**No**	96% (72/75)	78% (59/75)	<0.01

APF, arterial peak flow; ABI, ankle-brachial index; IC, intermittent claudication; CI, critical ischemia, PA, popliteal artery.

a Diabetic status could be determined in 166 out of 183 patients.

b In 5 out of 183 the PA or main arteries of the lower leg could not be assessed due to severe image artifacts caused by metallic knee prostheses.

**Table 3:** APF and ABI values and success rates in subgroups of patients.

	Intermittent claudication				Critical ischemia			
	Successful APF	APF (mL/s)	Successful ABI	ABI	Successful APF	APF (mL/s)	Successful ABI	ABI
**Diabetes**	95% (40/42)	5.7±3.5	79% (33/42)	0.72±0.22	95% (36/38)	4.6±3.0	61% (23/38)	0.57±0.21
**Non-Diabetes**	96% (43/45)	5.9±2.9	84% (38/45)	0.75±0.22	83% (34/41)	3.7±2.6	61% (25/41)	0.51±0.27

APF, arterial peak flow; ABI, ankle-brachial index.

### Factors influencing measurement success rate

There were large differences in the proportions of subjects in whom APF and ABI could be obtained between patients with intermittent claudication and critical ischemia (table 2, disease severity). The success rates were highest in patients with intermittent claudication (p = 0.06 and p = 0.01 for APF and ABI measurements, respectively) compared to those with critical ischemia.

As can be seen in table 3, approximately half of patients with diabetes mellitus in our population had intermittent claudication. In 17 patients the diabetic status could not be established definitively. These patients were referred to our institution for diagnostic purposes only and no full clinical history was present. Therefore, these patients were excluded from the analyses focused on diabetes. No significant differences in success rates were found between patients with and without diabetes for both APF and ABI measurements (p = 0.19 and p = 0.64 for APF and ABI measurements respectively). However, ABI measurements were significantly more often successful in diabetic patients with intermittent claudication (table 3, 84%) than those with critical ischemia (61%, p<0.01). No further significant differences were found between the subgroups of diabetes and disease severity in table 3.

Patients without arterial obstructive pathology below the level of the APF measurement showed a trend of higher success rates in APF (p = 0.07) and ABI (p = 0.11) measurements.

Main causes of failure of the APF measurements (n = 17, 9%) were hardware failure (n = 7), occlusion of the PA (n = 7) and irregular heart rate (n = 1). In 2 patients the cause of failure remained unknown.

Main causes of failure for ABI measurements (n = 53, 29%) were non-compressible arteries due to arterial stiffness (n = 42) and presence of PAD-related ulcers or wounds, making it impossible to use a cuff to measure the systolic pressure at the level of the ankle (n = 7). In 4 patients no cause of failure was reported in the patient records.

### Discriminative capability

**Figure 3:**
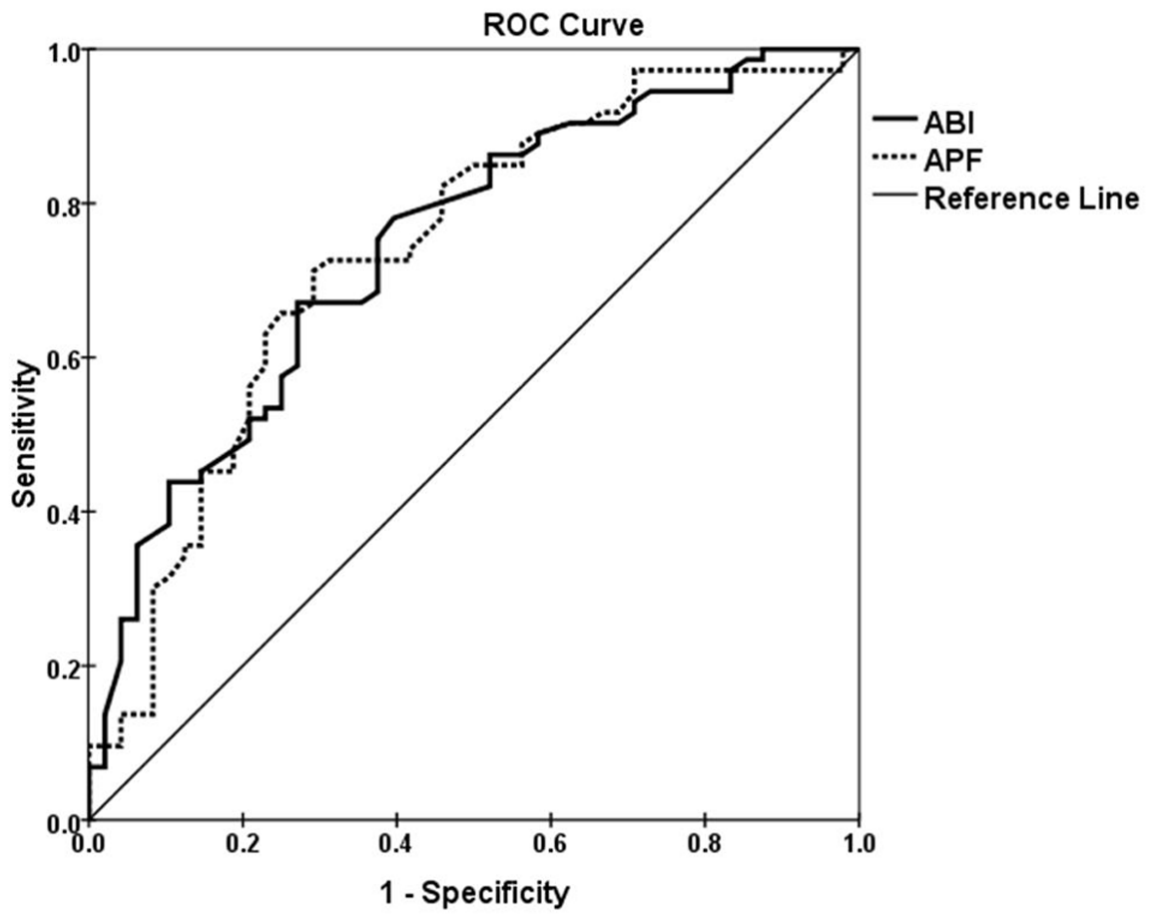
ROC curves for arterial peak flow (APF) and ankle-brachial index (ABI) in 122 patients with successful APF and ABI measurements. The curves show comparable discriminative capability for determination of disease severity (intermittent claudication versus critical ischemia) between APF and ABI measurements.

Figure 3 shows ROC curves for both APF and ABI measurements in 122 patients with intermittent claudication or critical ischemia, in whom both APF as well as ABI measurement were successfully obtained. The AUC of the APF was 0.74 (95% confidence interval (CI), 0.66 – 0.83) and 0.75 (95% CI, 0.65 – 0.83) for ABI, respectively, and were not statistically significant (p = 0.90).

## Discussion

Objective assessment of vascular function is important in the diagnostic work-up and follow-up of patients with PAD. The most widely used measure for this purpose is the ABI. Although the ABI has a high sensitivity to detect PAD, we found that the ABI could not be determined in up to 29% of the patients included in this study. Alternative functional measurements that provide information about peripheral vascular status would therefore be desirable to objectively characterize vascular status. The results of this study show that popliteal arterial peak flow (APF), measured with MRI in clinical PAD patients scheduled for MRA, has a success rate of up to 91%, which was significantly higher compared to ABI measurements (71%), with similar discriminative capability.

### Success rates

Although in 91% of the patients functional information could be obtained by performing APF measurements, combined measurement of ABI and APF resulted in a slight improvement to 96% (n = 176) in the proportion of patients with usable functional information. Therefore, APF measurements should not be considered as a replacement for ABI measurements, but rather as a useful addition.

ABI measurements were unsuccessful in up to 29% of the patients. This is markedly worse than the 5 – 10% known from previous literature [12], [15], [18]. The most likely explanation for this discrepancy is the specific population for this study, which comprises only PAD patients referred for a clinical CE-MRA exam, rather than the general population of PAD patients. This means our population is likely to harbor more severe disease compared to patients with PAD who do not require a CE-MRA for diagnosis or treatment planning. In this context, it should also be noted that other methods such as pulse volume recordings and digit pressures are not affected by non-compressible vessels or diabetes, and may serve as an additional tool to obtain functional information.

### Factors influencing success rate

ABI measurements could be determined more often in patients with intermittent claudication compared to patients with critical ischemia. This can be explained by the higher number of patients with ulceration and tissue loss as well as the more pronounced arterial stiffness in these patients with more severe state of PAD.

The presence of arterial lesions in the lower leg showed a trend towards lower success rates in both APF and ABI measurements. As far as the APF is concerned, this was due to the presence of popliteal occlusions, whereas for the ABI measurements this was due to the presence of skin ulcera/wounds and more pronounced arterial stiffness. As the APF is measured in the PA, while the ABI is acquired at ankle-level, a direct comparison between quantitative measures might not be reliable, especially in patients with arterial lesions in the lower leg. However, analysis of the APF data revealed significantly lower flow values for patients with significant arterial lesions in the lower leg. Therefore, flow measurements of the PA seem to be influenced by obstructive lesions in both in- and outflow trajectories. These results were concordant with the ABI data.

Another factor associated with a trend towards lower success rates of both APF and ABI measurements was the presence of arterial lesions at the level of and below the knee. In this patient group, APF could not be determined in case of popliteal occlusions. ABI measurements on the other hand, were hard to obtain in the patients with ischemic wounds and more pronounced arterial stiffness.

### Discriminative capability

Determination of APF with PCA has been shown to have good reproducibility and discriminative capabilities before [27]. In this study we found that APF in patients with critical ischemia was significantly lower compared to patients with intermittent claudication. AUCs of the ROC curves for both APF and ABI measurements were comparable. This indicates that there is no relevant difference in discriminative capability of APF and ABI measurements to distinguish PAD patients with intermittent claudication from those with critical ischemia. Therefore, APF measurements are a reliable alternative to ABI measurements in patients in whom the ABI cannot be determined.

### Arterial Peak Flow

Arterial peak flow, defined as the maximum systolic flow, is an attractive arterial flow measure. Although quantitative MRI-based flow measurements can also be used to measure mean flow, we chose to measure the APF only. This is an important determinant of the systolic blood pressure [28], that shows relatively large differences between PAD patients and control subjects and is more reproducible than the mean flow [29].

For this study only patients scheduled for MRA were included. Although a selected population was used, as far as APF measurements are concerned this is clinically the most relevant patient population, as MRI-based APF measurements are not suitable for screening purposes due to the limited availability of MRI systems or magnet time and associated costs. Also, as the selected study population comprised consecutive patients undergoing CE-MRA, it is a clinically representative group as far as disease severity and presence of diabetes is concerned. Nevertheless, this implies that current findings do not necessarily apply for all PAD patients. We do see an increasing number of PAD patients undergoing multiple MRA examinations during lifetime for treatment planning and/or therapy monitoring. A fast and simple functional MRI measurement, such as the APF, would therefore be a valuable addition to MRA in order to objectively quantify the vascular status and to monitor the progression of PAD over time, especially in patients in whom the ABI cannot reliably be determined.

This study and our previous work [27] demonstrate that the APF is a reproducible measure that can be acquired in almost every PAD patient scheduled for MRA and shows large differences between non-PAD patients and PAD patients with intermittent claudication or critical ischemia. Further research will be required to determine the most suitable location to assess the APF, to determine normative values and to demonstrate the clinical relevance of APF, ie. Its influence on therapy and clinical course of PAD.

## Conclusion

Compared to the assessment of PAD severity with ABI, the success rate of MRI-based APF measurements in patients with a clinical indication for MRA is 20% higher, with similar discriminatory capacity for disease severity. Therefore, APF is an especially convenient and valuable measure to assess severity in PAD patients scheduled for MR angiography to obtain additional functional information concerning the vascular status.

## References

[1] Hirsch AT, Haskal ZJ, Hertzer NR, Bakal CW, Creager MA, et al (2006) ACC/AHA 2005 guidelines for the management of patients with peripheral arterial disease (lower extremity, renal, mesenteric, and abdominal aortic): executive summary a collaborative report from the American Association for Vascular Surgery/Society for Vascular Surgery, Society for Cardiovascular Angiography and Interventions, Society for Vascular Medicine and Biology, Society of Interventional Radiology, and the ACC/AHA Task Force on Practice Guidelines (Writing Committee to Develop Guidelines for the Management of Patients With Peripheral Arterial Disease) endorsed by the American Association of Cardiovascular and Pulmonary Rehabilitation; National Heart, Lung, and Blood Institute; Society for Vascular Nursing; TransAtlantic Inter-Society Consensus; and Vascular Disease Foundation. J Am Coll Cardiol 47: 1239–1312. 16545667 10.1016/j.jacc.2005.10.009

[2] Norgren L, Hiatt WR, Dormandy JA, Nehler MR, Harris KA, et al.. (2007) Inter-Society Consensus for the Management of Peripheral Arterial Disease (TASC II). J Vasc Surg 45 Suppl S: S5–67. 10.1016/j.jvs.2006.12.037 17223489

[3] Gerhard-Herman M, Gardin JM, Jaff M, Mohler E, Roman M, et al (2006) Guidelines for noninvasive vascular laboratory testing: a report from the American Society of Echocardiography and the Society for Vascular Medicine and Biology. Vasc Med 11: 183–200. 17288127 10.1177/1358863x06070516

[4] Creager MA (1997) Clinical assessment of the patient with claudication: the role of the vascular laboratory. Vasc Med 2: 231–237. 9546973 10.1177/1358863X9700200312

[5] Andersen CA (2010) Noninvasive assessment of lower extremity hemodynamics in individuals with diabetes mellitus. J Vasc Surg 52: 76S–80S. 20804937 10.1016/j.jvs.2010.06.012

[6] Begelman SM, Jaff MR (2006) Noninvasive diagnostic strategies for peripheral arterial disease. Cleve Clin J Med 73 Suppl 4S22–29. 17385388 10.3949/ccjm.73.suppl_4.s22

[7] Aslam F, Haque A, Foody J, Lee LV (2009) Peripheral arterial disease: current perspectives and new trends in management. South Med J 102: 1141–1149. 19864983 10.1097/SMJ.0b013e3181bb9ab8

[8] McDermott MM, Criqui MH, Greenland P, Guralnik JM, Liu K, et al (2004) Leg strength in peripheral arterial disease: associations with disease severity and lower-extremity performance. J Vasc Surg 39: 523–530. 14981443 10.1016/j.jvs.2003.08.038

[9] McDermott MM, Greenland P, Liu K, Guralnik JM, Criqui MH, et al (2001) Leg symptoms in peripheral arterial disease: associated clinical characteristics and functional impairment. JAMA 286: 1599–1606. 11585483 10.1001/jama.286.13.1599

[10] Resnick HE, Lindsay RS, McDermott MM, Devereux RB, Jones KL, et al (2004) Relationship of high and low ankle brachial index to all-cause and cardiovascular disease mortality: the Strong Heart Study. Circulation 109: 733–739. 14970108 10.1161/01.CIR.0000112642.63927.54

[11] Doobay AV, Anand SS (2005) Sensitivity and specificity of the ankle-brachial index to predict future cardiovascular outcomes: a systematic review. Arterioscler Thromb Vasc Biol 25: 1463–1469. 15879302 10.1161/01.ATV.0000168911.78624.b7

[12] Potier L, Abi Khalil C, Mohammedi K, Roussel R (2010) Use and Utility of Ankle Brachial Index in Patients with Diabetes. Eur J Vasc Endovasc Surg. 10.1016/j.ejvs.2010.09.020 21095144

[13] Dachun X, Jue L, Liling Z, Yawei X, Dayi H, et al (2010) Sensitivity and specificity of the ankle–brachial index to diagnose peripheral artery disease: a structured review. Vasc Med 15: 361–369. 20926495 10.1177/1358863X10378376

[14] Guo X, Li J, Pang W, Zhao M, Luo Y, et al (2008) Sensitivity and specificity of ankle-brachial index for detecting angiographic stenosis of peripheral arteries. Circ J 72: 605–610. 18362433 10.1253/circj.72.605

[15] Aboyans V, Ho E, Denenberg JO, Ho LA, Natarajan L, et al (2008) The association between elevated ankle systolic pressures and peripheral occlusive arterial disease in diabetic and nondiabetic subjects. J Vasc Surg 48: 1197–1203. 18692981 10.1016/j.jvs.2008.06.005

[16] van Langen H, van Gurp J, Rubbens L (2009) Interobserver variability of ankle-brachial index measurements at rest and post exercise in patients with intermittent claudication. Vasc Med 14: 221–226. 19651671 10.1177/1358863X08101017

[17] Allen J, Oates CP, Henderson J, Jago J, Whittingham TA, et al (1996) Comparison of lower limb arterial assessments using color-duplex ultrasound and ankle/brachial pressure index measurements. Angiology 47: 225–232. 8638864 10.1177/000331979604700302

[18] Stein R, Hriljac I, Halperin JL, Gustavson SM, Teodorescu V, et al (2006) Limitation of the resting ankle-brachial index in symptomatic patients with peripheral arterial disease. Vasc Med 11: 29–33. 16669410 10.1191/1358863x06vm663oa

[19] Brooks B, Dean R, Patel S, Wu B, Molyneaux L, et al (2001) TBI or not TBI: that is the question. Is it better to measure toe pressure than ankle pressure in diabetic patients? Diabet Med 18: 528–532. 11553180 10.1046/j.1464-5491.2001.00493.x

[20] Giachelli CM (2004) Vascular calcification mechanisms. J Am Soc Nephrol 15: 2959–2964. 15579497 10.1097/01.ASN.0000145894.57533.C4

[21] Goss DE, de Trafford J, Roberts VC, Flynn MD, Edmonds ME, et al (1989) Raised ankle/brachial pressure index in insulin-treated diabetic patients. Diabet Med 6: 576–578. 2527696 10.1111/j.1464-5491.1989.tb01231.x

[22] London GM, Guerin AP, Marchais SJ, Metivier F, Pannier B, et al (2003) Arterial media calcification in end-stage renal disease: impact on all-cause and cardiovascular mortality. Nephrol Dial Transplant 18: 1731–1740. 12937218 10.1093/ndt/gfg414

[23] Raines JK, Darling RC, Buth J, Brewster DC, Austen WG (1976) Vascular laboratory criteria for the management of peripheral vascular disease of the lower extremities. Surgery 79: 21–29. 1246689

[24] de Vries M, de Koning PJ, de Haan MW, Kessels AG, Nelemans PJ, et al (2005) Accuracy of semiautomated analysis of 3D contrast-enhanced magnetic resonance angiography for detection and quantification of aortoiliac stenoses. Invest Radiol 40: 495–503. 16024987 10.1097/01.rli.0000163004.65460.8b

[25] de Vries M, Nijenhuis RJ, Hoogeveen RM, de Haan MW, van Engelshoven JM, et al (2005) Contrast-enhanced peripheral MR angiography using SENSE in multiple stations: feasibility study. J Magn Reson Imaging 21: 37–45. 15611941 10.1002/jmri.20240

[26] Leiner T, Kessels AG, Nelemans PJ, Vasbinder GB, de Haan MW, et al (2005) Peripheral arterial disease: comparison of color duplex US and contrast-enhanced MR angiography for diagnosis. Radiology 235: 699–708. 15858107 10.1148/radiol.2352040089

[27] Versluis B, Backes WH, van Eupen MG, Jaspers K, Nelemans PJ, et al (2011) Magnetic resonance imaging in peripheral arterial disease: reproducibility of the assessment of morphological and functional vascular status. Invest Radiol 46: 11–24. 21102349 10.1097/RLI.0b013e3181f2bfb8

[28] Bishara RA, Taha W, Alfarouk MO, Abdel Aal K, Wasfy S (2004) Duplex detected ankle peak systolic velocity: a new parameter for the assessment of degree of peripheralischemia. Int Angiol 23: 368–372. 15767982

[29] Mohajer K, Zhang H, Gurell D, Ersoy H, Ho B, et al (2006) Superficial femoral artery occlusive disease severity correlates with MR cine phase-contrast flow measurements. J Magn Reson Imaging 23: 355–360. 16463304 10.1002/jmri.20514

[30] Leiner T, Nijenhuis RJ, Maki JH, Lemaire E, Hoogeveen R, et al (2004) Use of a three-station phased array coil to improve peripheral contrast-enhanced magnetic resonance angiography. J Magn Reson Imaging 20: 417–425. 15332249 10.1002/jmri.20129

[31] Prakash A, Garg R, Marcus EN, Reynolds G, Geva T, et al (2006) Faster flow quantification using sensitivity encoding for velocity-encoded cine magnetic resonance imaging: in vitro and in vivo validation. J Magn Reson Imaging 24: 676–682. 16878307 10.1002/jmri.20654

[32] Hanley JA, McNeil BJ (1983) A method of comparing the areas under receiver operating characteristic curves derived from the same cases. Radiology 148: 839–843. 6878708 10.1148/radiology.148.3.6878708

